# Clinical and psychosocioeconomic impact of COVID-19 pandemic on patients of the Indian Progressive Systemic Sclerosis Registry (IPSSR)

**DOI:** 10.1093/rap/rkab027

**Published:** 2021-04-23

**Authors:** Chengappa Kavadichanda, Vineeta Shobha, Parasar Ghosh, Anupam Wakhlu, Devender Bairwa, Manju Mohanan, Ramya Janardana, Geetabali Sircar, Rasmi Ranjan Sahoo, Sneha Joseph, Vir Singh Negi, Dinesh Khanna, Padmanabha Shenoy

**Affiliations:** 1 Department of Clinical Immunology, Jawaharlal Institute of Postgraduate Medical Education and Research, Puducherry; 2 Department of Clinical Immunology and Rheumatology, St. John’s Medical College Hospital, Bengaluru, Karnataka; 3 Department of Rheumatology and Clinical Immunology, Institute of Post-Graduate Medical Education & Research and S. S. K. M. Hospital, Kolkata, West Bengal; 4 Department of Clinical Immunology and Rheumatology, RALC Campus, King George's Medical University, Lucknow, Uttar Pradesh; 5 Centre for Arthritis and Rheumatism Excellence (CARE), Cochin, Kerala, India; 6 Division of Rheumatology, Department of Internal Medicine, Scleroderma Program, University of Michigan, Ann Arbor, MI, USA

**Keywords:** rheumatic diseases, cohort, scleroderma, mental health, health economics

## Abstract

**Objectives:**

The aim was to determine the impact of the coronavirus disease 2019 (COVID-19) pandemic on access to health care among patients with scleroderma and to analyse the economic and psychosocial impacts and the infection prevention measures taken by them during the pandemic.

**Methods:**

A 25-item questionnaire designed to assess the components of the objectives was tele-administered between October 2020 and January 2021 to the patients enrolled in the Indian Progressive Systemic Sclerosis Registry.

**Results:**

Of the 428 patients in the registry, 336 took part in the study. A scheduled outpatient visit was missed by 310 (92.3%) patients, and 75 (22.3%) skipped prescription drugs. During the pandemic, 75 (22.3%) had a family member lose a job. Financial difficulties were reported by 155 (46.1%), with 116 (34.5%) patients having to spend an additional INR 4000 (2000–10 000) [USD 54.9 (27.0–137.4)] to continue treatment. Although 35 patients (10.4%) had at least one symptom suggestive of COVID-19, infection was confirmed in only 4. None of them needed hospitalization or had adverse outcomes. Worsening of scleroderma was seen in 133 (39.6%) individuals, with 15 (4.5%) requiring hospitalization. Most (96%) of the patients were aware of infection prevention measures, and 91 (27.1%) had taken unproven prophylactic medications.

**Conclusion:**

Individuals with scleroderma in India have been affected during the pandemic owing to closure of hospital services, lack of transport, loss of jobs and the additional financial burden. Health-care providers should continue to educate patients to stay on their medications and encourage them to be vaccinated for COVID-19.

Key messages

- Most patients with scleroderma missed their scheduled hospital visits during the coronavirus disease 2019 pandemic.
- Coronavirus disease 2019 was rare and mild among patients with scleroderma in India.
- Devising mechanisms to ensure continuity of care and address mental health issues will help in making scleroderma programmes future proof.

## Introduction

The ongoing COVID-19 pandemic has brought to scrutiny the strengths and weaknesses of health-care systems across the globe [1, 2]. The common theme emerging from this scrutiny is that most systems across the world are not capable of identifying and addressing the needs of susceptible groups of individuals who require protection and specialized care during times of crisis. Susceptible groups such as the elderly and those with chronic diseases including psychiatric illness [3], malignancies [4] and autoimmune inflammatory rheumatic diseases (AIRDs) [5, 6] have been badly affected during this pandemic. Given the abrupt changes caused by the pandemic and the mitigation measures imposed by governments, individuals who have poor physical and functional status are likely to be affected the most.

AmongAIRDs, scleroderma or SSc has a very high rate of mortality and morbidity [7, 8], with patients having poor physical and functional status. Individuals with AIRDs, including those with SSc, require regular and periodic clinical assessment by rheumatologists and laboratory check-ups for the early detection of disease worsening or other complications. SSc is characterized by skin thickening, vasculopathy and damage to the digits and internal organs, especially lungs, heart and gastrointestinal tract, leading to severe physical and psychological impairment [9].

With India announcing a strict nationwide lockdown from 23 March 2020 to 31 May 2020 to contain the spread of COVID-19, followed by a gradual opening up of services from 1 June 2020 onward [10], most of the patients with chronic diseases were deprived of access to routine health care. In order to overcome the challenges in health-care delivery during the pandemic, governments and health-care providers have devised and rapidly implemented several simple and sustainable strategies [11]. Introduction of telemedicine and online consultations is one such strategy that has helped many chronically ill patients, including those with SSc. Tele-rheumatology has not only helped patients seek timely health care but has also enabled clinicians to manage patient cohorts effectively and continue research in a modified manner [12]. The data generated by the tele-rheumatology consultations and questionnaire-based surveys will be useful in preparing for further catastrophes and addressing myths and beliefs among patients and their care providers. In this context, limited studies have been done to understand the accessibility and availability of drugs used in rheumatology practice and health-care services during the nationwide lockdown. Also, there have been limited attempts to understand the challenges faced, health beliefs and practices of patients with SSc.

The aim of the present study was to determine the health-care impact of the COVID-19 pandemic by tele-consultation and the issues faced in continuing medications among the patients enrolled in the Indian Progressive SSc Registry (IPSSR). We also analysed the psychosocial impact of the COVID-19 pandemic on scleroderma patients and sought to understand the measures taken by patients with SSc to protect themselves from the severe acute respiratory syndrome coronavirus 2 (SARS-CoV-2) infection during the peak of the pandemic during 2020 in India.

## Methods

The IPSSR is an ongoing multicentre cohort of patients with SSc in India. Six centres in different parts of India are involved in recruiting and following up patients with SSc as a part of this registry, namely: Centre for Arthritis and Rheumatism (CARE), Cochin, Kerala; Jawaharlal Institute of Postgraduate Medical Education and Research, Puducherry; Department of Rheumatology and Clinical Immunology, St. John’s Medical College Hospital, Bengaluru, Karnataka; Institute of Post-Graduate Medical Education & Research and S. S. K. M. Hospital, Kolkata, West Bengal; Department of Rheumatology, RALC Campus, King George's Medical University, Lucknow, Uttar Pradesh; and Sanjay Gandhi Post Graduate Institute of Medical Sciences, Lucknow, Uttar Pradesh. Patients who fulfil the 2013 ACR/EULAR classification criteria for SSc [13] have been recruited into the cohort prospectively since March 2019 and are followed up at 3-month intervals across all the centres.

Institute ethics approval was obtained by all the participating centres. The present telemedicine-based study was designed to establish contact with the patients enrolled in the registry during the time of COVID-19. A questionnaire with 25 items was developed in the English language by two experienced rheumatologists (Supplementary Data S1, available at *Rheumatology Advances in Practice* online). These questions were then discussed with the investigators from all the participating centres and finalized. The questions covered issues on health-care delivery, medical complications during the lockdown, COVID-19-related symptoms, if any, preventive measures practised by the patients and psychosocial issues. The questionnaire was then administered in the vernacular languages to the research team by the rheumatologists to test for interlanguage reliability with the answers obtained [14]. After ascertaining the reliability of the questions, all the enrolled patients were contacted between October 2020 and January 2021on their registered mobile/telephone numbers and email addresses. Those patients who consented to participate in the study were administered the questionnaire by a trained speciality nurse over the telephone, WhatsApp, email or a video consultation that was supervised by a rheumatologist. Verbal consent was obtained from the patients before administration of the questionnaire, and waiver of written consent was obtained because this was a telemedicine-based study.

The medical officer explained the questions along with all the options to the patient in English or their vernacular language (Malayalam, Tamil, Hindi, Bengali, Kannada or Telugu), and the feedback was documented manually on printed forms. The patient global assessment was done using the Likert scale from 0 to 10, where 0 meant feeling worst and 10 meant very good. The participants were not offered any type of incentive for participating in the study. Once the questionnaire was filled, a completeness check and a multiple entries check was done by manually screening the entries by the study nurse. This was followed by telephonically assessing the scleroderma health-associated questionnaire (SHAQ) and hospital anxiety depression scale (HADS). A HADS score of above seven was considered abnormal for depression and anxiety separately [15]. The patients were also allowed to revise their previous responses if required during this call [16]. The data were then entered manually into an Excel sheet, and only questionnaires completely filled were analysed.

### Statistical methods

The data were analysed using IBM SPSS Statistics v.19 software. Continuous variables were represented as the mean (s.d.) or the median and interquartile range (IQR) or range, as appropriate. The Kolmogorov–Smirnov test was used to check the normality of the quantitative data. Categorical variables such as sex, disease status, presence of co-morbidities, compliance, availability of drugs, COVID-19-related symptoms and knowledge of precautions, a questionnaire assessing physical and mental health, were described as frequencies and proportions. Variables determining abrupt stoppage of prescription drugs, flare of SSc symptoms and abnormal HADS score were compared using chi-square or the Mann–Whitney *U*-test. A *P*-value of <0.05 was considered as significant.

## Results

### Baseline characteristics

The speciality nurse from each study centre attempted to contact 428 patients. Of the 428 calls, 338 patients responded and 336 consented to participate in the study. The demographic, clinical and treatment details of the participating patients are presented in Table 1. The female-to-male ratio of the patients in the cohort was 12.4:1, with a duration of illness ranging from 24 to 96 months and a median of 48 months. Of the 336 patients who participated in the study, 189 (56.3%) had a diffuse cutaneous SSc phenotype and 123 (36.6%) had limited cutaneous SSc. Data about the subtype of SSc were not available for 11 (3.3%) patients. The average monthly income of the families of the patients was INR 10 000 (IQR 5000–20 000) [USD 137.4 (IQR 68.7–274.8)].

**Table 1:** Baseline demographic and clinical details of the participants enrolled in the study

Parameter (*n* = 336)	Number (%)/mean (s.d.)
Age, mean (s.d.), years	42 (11.6)
Female, *n* (%)	311/336 (92.6)
Male, *n* (%)	25/336 (7.4)
Duration of disease, mean (s.d.), months	48 (24–96)
HAQ-DI	0.69 (0.2–1.1)
Disease subtype, *n* (%)	
Diffuse	189/336 (56.3)
Limited	123/336 (36.6)
Sine scleroderma	13/336 (3.9)
Unknown	11/336 (3.3)
Occupation, *n* (%)	
Housewife	249/336 (74.1)
Employed	56/336 (16.7)
Unemployed	7/336 (2.1)
Student	23/336 (6.8)
Monthly family income (USD)	137.4 (range 34.4–1374.4)
**Co-morbidities, *n* (%)**	
Hypertension	41/336 (12.20)
Ischaemic heart disease	3/336 (0.89)
Diabetes mellitus	18/336 (5.38)
Hypothyroidism	57/336 (16.96)
Others	7/336 (2.08)
**Treatment, *n* (%)**	
MTX	64/270 (23.7)
LEF	33/365 (4.2)
Tacrolimus	4/266 (1.5)
MMF	79/266 (29.7)
HCQ	86/250 (25.6)
CYC	9/267 (3.4)
Tadalafil	179/310 (57.7)
Aspirin	22/267 (8.2)
Other medicines	281/336 (83.6)

HAQ-DI: health associated questionnaire disability index; USD: United States dollars. The results are presented as the mean (s.d.) or median with interquartile range, as appropriate, along with the number of patients in the analysis.

### Logistics and health-care-related challenges during the pandemic

**Fig. 1:**
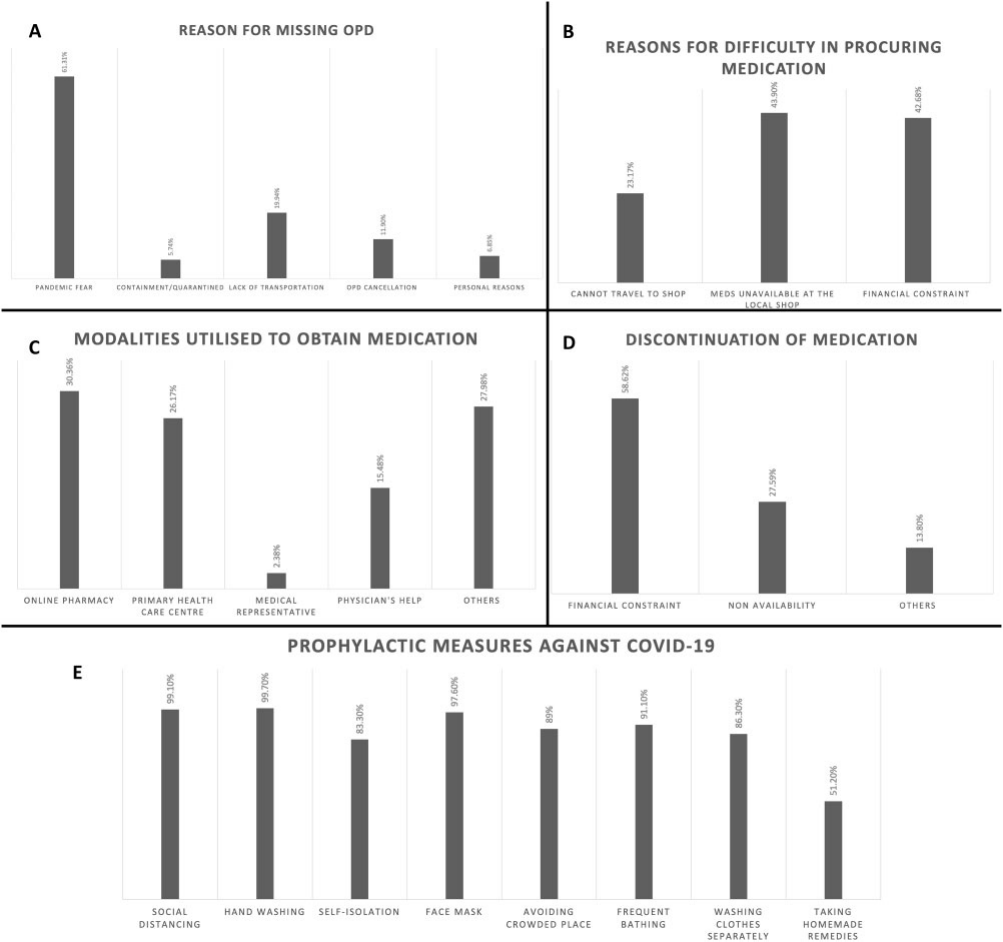
Challenges faced by patients in accessing health care during the pandemic and practices to overcome them (**A**) Reasons for missing outpatient visit. (**B**) Reasons for difficulty in procuring drugs for SSc. (**C**) Alternative methods adopted to procure medications. (**D**) Reasons for discontinuation of drugs. (**E**) Preventive measures adopted by patients with SSc against coronavirus disease 2019 (COVID-19) infection.

We found that 310 (92.3%) patients missed their scheduled outpatient department (OPD) visit, and 75 (22.3%) had to skip their prescription medications. Fofty-two patients (15.4%) skipped the medications for ≤1 month, whereas 23 patients (6.9%) skipped medications for >1 month. The reasons for missing the OPD visits were pandemic fear, lack of transportation or OPD cancellation, among others, and the reasons for skipping the prescription drugs were financial constraints and non-availability (Fig. 1). Among those who managed to continue the medications (*n* = 272) despite the lockdown and containment measures, the various modalities by which they obtained the drugs were online pharmacy, primary health-care centres, doctor’s help, medical representatives and accredited social health activists (Fig. 1). During the lockdown, 185 (55.1%) patients were contacted and assisted by their health teams before the current call, and the commonest mode of contact was telephone call, followed by WhatsApp mobile App and video call.

Owing to the lockdown measures, 75 (22.3%) families had a family member lose their jobs and 155 (46.1%) faced financial difficulties (Table 2). Besides these, 116 (34.5%) patients had to spend extra cash to obtain treatment for SSc, and the average extra amount spent was INR 4000 (range 2000–10 000) [USD 54.9 (range 27.5–137.4)].

**Table 2:** The impact ofcoronavirus disease 2019 pandemic on health-care accessibility, scleroderma disease activity and psychosocial aspects

Health-care accessibility-related issues	**Number (%)** **(total *n* = 336)**
Missed OPD appointment	310 (92.3)
Missed laboratory testing	179 (53.3)
Faced problems with availability of medicines	82 (24.4)
Discontinued medications	75 (22.3)
Time period for which medication discontinued	
<10 days	27 (8.0)
10–30 days	25 (7.4)
30–60 days	8 (2.4)
>60 days	15 (4.5)
Contacted by medical team members before the study	185 (55.1)
Telephone	173 (51.49)
WhatsApp	18 (5.36)
Video consultation	4 (1.19)
Others	64 (19.05)
Worsening of SSc symptoms	133 (39.6)
RP	91 (27.1)
Digital/non-digital ulcers	63 (18.8)
Skin tightening	82 (24.4)
Joint pain	120 (35.7)
Others	56 (16.7)
Hospital visit owing to increased disease activity	81 (24.1)
Hospitalized during pandemic	15 (5.4)
**Psychosocioeconomic factors**	
Loss of job owing to pandemic	75 (22.3)
Financial difficulty	155 (46.1)
Money expenditure more than routine	116 (34.5)

OPD: outpatient department.

### COVID-19-like manifestations and preventive measures

Between March 2020 and December 2020, 35 patients (10.4%) had at least one symptom of COVID-19, which included fever (*n* = 27; 8.0%), cough (*n* = 28; 8.3%) and new-onset or worsening breathlessness (*n* = 24; 7.1%); two patients developed diarrhoea, and three patients had anosmia. Testing for COVID-19 was done for 59 patients (17.6%), and real-time RT-PCR confirmed infection in four patients. None of the SSc patients with the SARS-CoV-2 infection needed hospitalization, and at the time of the call they had completely recovered from it (Table 3). Most (>96%) of the contacted patients were aware of preventive measures against COVID-19 infection, such as maintaining social distancing, frequent hand washing, isolation and using a face mask (Fig. 1E). It was noticed that 91 patients (27.1%) had taken unproven prophylactic medications to prevent themselves from COVID-19 infection. These prophylactic medications or supplements included ayurvedic medicines (*n* = 37), homeopathic medicines (*n* = 48) or remedies such as zinc (*n* = 30; 8.9%), vitamin C (*n* = 39; 11.6%) and vitamin D (*n* = 09; 2.7%). Although some patients with SSc followed these preventive measures, 146 patients (43.5%) were apprehensive that their pre-existing diseases might flare up, and 140 patients (41.7%) felt that they had a higher risk of getting the infection.

**Table 3:** Occurrence of coronavirus disease 2019symptoms, confirmed infection, beliefs and practices surrounding aspects of self-care during pandemic

**Symptoms and behaviours**	Number (%) (total *n*=336)
Developed any symptoms suggestive of COVID-19	35 (10.4)
Fever	27 (8.0)
Breathlessness	24 (7.1)
Cough	28 (8.3)
Anosmia	3 (0.9)
Diarrhoea	2 (0.6)
Tested for SARS-CoV-2	59 (17.6)
RT-PCR testing	30 (08.9)
Antigen testing	31 (09.2)
Test confirmed COVID-19 infection	4 (1.2)
Mingled with COVID-19-positive patients	7 (2.1)
Family member was positive for COVID-19	8 (2.4)
Family members were exposed to COVID-19-positive patients	31 (9.2)
**Beliefs and fears**	
Do you think your disease might flare up owing to COVID-19?	
Yes	146 (43.5)
No	190 (53.6)
Do you think you are more likely to become infected with COVID-19?	
Yes	140 (41.7)
No	196 (58.3)
Do you feel more vulnerable to COVID-19?	
Very much	69 (20.5)
Mild	105 (31.3)
Not at all	162 (48.2)
What is the cause of your increased vulnerability to COVID-19?	
Disease	144 (42.9)
Drugs	17 (5.1)
Other	None

COVID-19: coronavirus disease 2019; SARS-CoV-2; severe acute respiratory syndrome coronavirus 2.

### Impact of COVID-19 on SSc and mental health

We analysed the characteristics of the patients who had worsening of SSc symptoms during the lockdown (Table 4). One hundred and thirty-three patients experienced worsening of their scleroderma symptoms during the pandemic, and the details of the symptoms are presented in Table 2. Of these patients, 15 needed hospitalization.

**Table 4:** Comparison of various factors between those who had worsening of disease activity *vs* those who remained stable during the pandemic

Variable	Worsening of SSc (*n* = 133)	No worsening of SSc (*n* = 201)	*P*-value
Monthly family income, INR [USD]	10 000 (IQR 5000–18 000) [137.1 (68.5–246.7)]	10 000 (IQR 6000–20 000) [137.1 (82.2–274.12)]	0.39
Disease duration, months	54 (IQR 36–96)	36 (IQR 12–69)	0.75
Extra expenditure	3000 (IQR 2000–6750)	5000 (IQR 3000–10 000)	0.25
Financial difficulty, *n* (%)	62 (46.6)	93 (45.8)	0.49
Job loss, *n* (%)	43 (32.3)	32 (15.8)	<0.01
HAQ score (0–3)	0.88 (IQR 0.2–1.9)	0.50 (IQR 0.13–1.1)	0.15
PGA (0–10 scale)	5 (IQR 3.2–7.0)	3 (IQR 2–4.7)	<0.01
Drug discontinued,^a^*n* (%)	38 (38.5)	37 (19.9)	0.01
Missed OPD appointment, *n* (%)	126 (94.7)	184 (91.1)	0.15
Symptoms suggestive of COVID-19, *n* (%)	13 (9.8)	22 (10.8)	0.45
COVID-19 positivity, *n* (%)	2 (1.5)	2 (1)	0.35
Family tested positive for COVID-19, *n* (%)	7 (9.7)	1 (0.5)	0.01
Extra financial expenditure, *n* (%)	47 (35.6)	69 (34)	0.43

COVID-19: coronavirus disease 2019; HAQ: Health associated questionnaire; INR: Indian rupee; IQR: interquartile range; OPD: outpatient department; PGA: physician global assessment; USD: United States dollars. ^a^Data available for only 284 (98 worsening disease *vs* 186 no worsening).

Patients who had discontinued medications and those under psychological stress owing to loss of employment had a higher association with worsening of SSc-related symptoms (*P* < 0.01). One hundred and fourteen patients had abnormal scores (>7) for depression and 149 for anxiety. Furthermore, upon analysing the patients who had an abnormal (>7) depression and anxiety score on HADS, we found that people who had experienced a job loss or had financial difficulty and those who had worsening SSc symptoms were more likely to have abnormal HADS scores (*P* < 0.01; Table 5).

**Table 5:** Factors associated with abnormal hospital anxiety depression scale score among patients with scleroderma

Variable	HADS-D > 7 (*n* = 114)	HADS-D ≤ 7 (*n* = 222)	*P*-value	HADS-A > 7 (*n* = 149)	HADS-A ≤ 7 (*n* = 187)	*P*-value
Monthly family income, INR [USD]	10 000 (IQR 5000– 18 000) [137.1 (68.5–246.7)]	10 000 (IQR 6000– 20 000) [137.1 (82.2–274.12)]	<0.01	8000 (IQR 5000– 15 000) [109.6 (68.5–205.6)]	10 000 (IQR 7000– 20 000) [137.1 (95.9–274.12)]	0.27
Financial difficulty, *n* (%)	63 (55.3)	92 (41.4)	0.01	71 (47.7)	84 (44.9)	0.35
Job loss, *n* (%)	39 (34.2)	36 (16.2)	<0.01	45 (30.2)	30 (16)	<0.01
Extra expenditure during pandemic, *n* (%)	52 (46)	69 (28.8)	<0.01	60 (40.5)	56 (29.9)	0.03
Disease duration, months	54 (IQR 36–96)	36 (IQR 12–69)	0.31	48 (IQR 36–96)	24 (IQR 12–72)	0.27
Missed OPD, *n* (%)	106 (93)	204 (92.3)	0.51	126 (94.7)	184 (91.1)	0.15
Drug discontinued, *n* (%)	30 (26.3)	34 (15.3)	0.01	30 (22.6)	34 (16.7)	0.12
HAQ score (0–3)	0.88 (IQR 0.2–1.9)	0.50 (IQR 0.13–1.1)	0.05	0.63 (IQR 0.2–1.9)	0.50 (IQR 0.16–1.08)	<0.01
PGA (0–10 scale)	5 (IQR 3.2–7.0)	3 (IQR 2–4.7)	<0.01	5 (IQR 3–5.75)	3 (IQR 2–4.7)	<0.01
Scleroderma symptoms worsened, *n* (%)	64 (56.1)	69 (31.1)	<0.01	82 (55)	51(27.3)	<0.01
Hospital visit, *n* (%)	22 (19.3)	459 (26.6)	0.85	33 (22.1)	48 (25.7)	0.72
Hospitalized during pandemic, *n* (%)	12 (10.5)	23 (10.4)	0.55	12 (8.1)	06 (3.2)	0.02
Symptoms suggestive of COVID-19, *n* (%)	13 (9.8)	22 (10.8)	0.45	17 (11.4)	18 (9.6)	0.45

COVID-19: coronavirus disease 2019; HADS: hospital anxiety depression scale; HAQ: health associated questionnaire; INR: Indian rupee; OPD: outpatient department; PGA: physician global assessment; USD: United States dollars.

## Discussion

In the present study, we analyse the impact of the COVID-19 pandemic on individuals with SSc who are enrolled in a nationwide multicentre registry. It is well established that telemedicine is a widely accepted modality for obtaining health care among patients with AIRDs, both in India [17] and in other parts of the world [18, 19]. It is interesting to note that even during the peak of COVID-19 transmission during the periods between July and November 2020 in various parts of India, only 4 (1.2%) patients out of 336 contracted the infection. Studies conducted before the 2020 peak of COVID-19 in India also reported low rates of COVID-19 infection among patients with SLE [12, 20]. However, we must interpret the results of our study with caution, because the status and outcome of individuals who were not reachable by the medical teams are not yet known. Moreover, the infection was cured without any unfavourable outcomes in all these patients, even though all of them were on immunosuppressant medications, reaffirming the fact that patients with AIRDs might have similar outcomes from COVID-19 infection to the general population [21].

The present telemedicine-based study shows that the pandemic and the associated containment measures imposed substantial logistic and financial burdens on the patients and their families. In the cohort, we observed that 22% of the patients had at least one member in the family who lost their job. Close to 50% of the families of patients faced financial difficulties. The result of these disruptions seems to be direct, with >90% of the patients with SSc missing their scheduled OPD visit and >20% having to discontinue their drugs. This was similar to the reports from other parts of the world, where more than two-thirds of patients reported disruption of scleroderma treatment services. The reason for discontinuing the drugs was mainly financial challenges faced by the families and the unavailability of drugs, which was also reported in the survey conducted by EURORDIS-Rare Diseases Europe [22]. Besides drugs, patients with SSc often have periodic assessments, such as pulmonary function tests and echocardiograms, involving doctors from other specialities, all of which have been discontinued during the pandemic [23]. The majority of the SSc patients switched to primary health-care centres and online pharmacies to procure drugs, because they could not attend the tertiary care hospitals during the pandemic. These are encouraging trends showing that patients are resourceful and motivated, which will go a long way in future preparedness. Another important finding of our study is that psychological stressors, such as job loss, along with obvious reasons, such as discontinuation of medication, were associated with worsening of SSc symptoms. In a previous telemedicine-based study, we had reported that patients could greatly reduce their out-of-pocket expenditure if the facilities for drug distribution were optimized at the primary health-care centre closest to their residence [24]. These results suggest that optimizing online pharmacies and pharmacies at primary health-care centres can help patients with chronic diseases both financially and psychologically.

Earlier studies have shown that patients with SSc changed their lifestyle greatly during the pandemic, adopting to more restrictive isolation measures, because of their awareness of frailty [25]. A similar behaviour was self-reported in our cohort, with the majority (>95%) of them abiding by the strict infection prevention practice, such as maintaining social distancing, wearing masks, isolating themselves and avoiding stepping outside the house. Most patients also practised some unproven infection mitigation measures, such as washing the clothes of the person stepping out of the house in a separate batch (80%) and having a bath after getting back home if she/he ventured out, besides taking prophylactic complementary alternative medicines and vitamins. It is yet to be determined whether these behavioural changes were a result of fear of the pandemic or owing to the sense of being frail among patients of AIRDs. Several symptoms, such as cough, dyspnoea and diarrhoea, that qualify for screening of COVID-19 infection were seen among patients with SSc but were not attributable to COVID-19. A similar incidence of probable and definite symptoms of COVID-19 infection among patients with SSc has also been reported from Italy [26]. Most of these symptoms are not specific for COVID and can also be seen with worsening disease activity in SSc. It is important for the physicians treating COVID-19 to screen these patients judiciously for infections and fast-track the referral of these patients to a specialist if negative for infection. This will help the rheumatologist to optimize treatment for SSc, thus preventing long-term complications. More than 30% of patients had abnormal scores for depression and anxiety. The prevalence of abnormal scores in patients with rheumatic diseases is similar to that reported from other countries [27]. Our study shows an association between worsening of SSc symptoms not only with drug discontinuation but also with psychological stressors, such as job loss and family members being diagnosed with COVID-19. This highlights the need to take a holistic approach and to include management of mental health problems among these patients. These psychosocioeconomic and clinical factors seem to be reflected in the higher association with depression and anxiety scores among our patients. Although we do not have a pre-COVID-19 value of HADS, the factors associated with higher scores show the high indirect morbidity in SSc during the pandemic. Similar results were also shown by a global e-survey of individuals with SSc [28] and a Web-based survey from India, which showed higher levels of anxiety among patients with AIRDs [29].

Our study has a few limitations, in that the pre-COVID-19 health and psychological status of these patients was not included in this study. Secondly, we could not reach nearly one-quarter of the patients for to various reasons, which is a drawback of conducting survey-based research. Besides these, being a questionnaire-based survey, the study might have response bias, whereby the individual might have over- or under-scored the questions. But there are a few strengths that could negate these shortcomings. The study involved a large cohort of SSc patients from different strata of society and different geographical locations across India, also reflected by similarity in demographic characteristics previously reported by a different group [30]. Moreover, very few other studies have documented the experience of patients during and after the 2020 peak of COVID-19 infection.

Our observations show that the individuals with SSc seem to have been affected during the pandemic on three fronts: (a) the constant fear of contracting COVID-19, resulting in a poor outcome as a consequence of the pre-existing condition or medications; (b) the difficulty in accessing health care owing to the abrupt change in the access, quality and the way routine health care is delivered during the pandemic; and (c) the economic crisis and loss of employment owing to the pandemic.

These findings highlight the fact that health-care providers should continue to educate patients to stay on their medications and encourage them to get vaccinated for COVID-19 infection. A close and complementary working between governmental agencies and private players to ensure continuity of care, availability of drugs and optimization of teleconsultation for specialist opinions will help us to mitigate these challenges to a large extent. Besides addressing these aspects, mechanisms to address the mental health issues need to be devised urgently to help patients with SSc cope with the circumstances. Such working models can be extrapolated to the care of most of the chronic diseases and can go a long way toward strengthening our health-care system.

## Supplementary Material

rkab027_Supplementary_Data
