# Supplementary material for: Clinical and psychosocioeconomic impact of COVID-19 pandemic on patients of the Indian Progressive Systemic Sclerosis Registry (IPSSR)
Source: Rheumatol Adv Pract. 2021 Apr 23;5(2):rkab027. doi: 10.1093/rap/rkab027 (PMC8135468; doi:10.1093/rap/rkab027)
Supplement: rkab027_Supplementary_Data [file rkab027_supplementary_data.docx]

**Supplementary Data S1:**

| Patient Name: ______________________ OPD/MRD/ Subject ID: _________________ Centre/Inv name: _____________  Tel: __________________________ Email Id: __________________________  Occupation: Housewife/unemployed/ Employee Monthly family income (INR): ________________________ |
| --- |

PSS-COVID Survey

Age at Diagnosis (Yrs.): _________ Sex: M/F

Current Age (Yrs.): _____ ACR/EULAR score: ______

Type:  Sine  Limited  Diffuse

| Clinical Features: (Circle as appropriate) | | | | | | | | | | | | | | | |
| --- | --- | --- | --- | --- | --- | --- | --- | --- | --- | --- | --- | --- | --- | --- | --- |
| Overlap SSc | RA | SLE | | | IIM | | Sjogrens | | | | Others: | | | | None |
| Pulmonary/ Cardiac Disease | | | | | | | | | PAH | | | | ILD   \| NSIP \| UIP \| Others \| \| --- \| --- \| --- \| | | None |
| Renal Disease | | | | Scl. Renal crisis | | | | | | Chronic renal failure | | | | Acute renal failure | None |
| Co-Morbid states | HTN | | IHD | | | DM | | Thyroid | | | | COPD/Asthma/ others……………….. | | | None |

Current Medication (Tick as appropriate)

| Steroid (Prednisolone Dose_:………. | | HCQ |
| --- | --- | --- |
| Methotrexate | Leflunomide | Tacrolimus |
| MMF | Cyclophosphamide | ACE/ARB inhibitors |
| AZR | Biologics | Tadalafil |
| Aspirin | Anticoagulant | Others:…………………… |
| Did your Medication get discontinued  Yes  No  IF yes, reason? -financial constraints/unavailability/others:…………………………………………….  Time period in which skipped meds?  1-10 days  10-30 day  30-60 days  >60 days | | |

| Health Care Delivery Issues | | | | | | | | | |
| --- | --- | --- | --- | --- | --- | --- | --- | --- | --- |
| 1. | Did you face medicine unavailability?  Yes  No  If yes reason: - Can’t travel to shop /No meds available in local Shops/Financial constraints | | | | | | | | |
| 2. | How did you managed to get medicine?  online pharmacy/primary health centers/medical representative/doctors help/Asha workers/others. | | | | | | | | |
| 3. | Did you have medication schedule change due to unavailability?  Specify the medication change made: | | | | Totally changed | | Mild change | | No change |
| 4. | Which Medicines were difficult to get? | HCQs | MMF | Tadalafil | | Steroids | | Others:__________ | |
| 5. | Did you miss scheduled OPD visits?  Yes  No  If yes, Reason: -pandemic fear/ Containment zone/quarantined/ Personal reasons/ Transportation difficulty due to pandemic/OPD cancellation by hospital. | | | | | | | | |
| 6. | Was there difficulty in Lab tests execution?  Yes  No  If yes, Reason: -pandemic fear/ Containment zone/quarantined/Financial constraints/  Transportation difficulty due to pandemic/No labs nearby/Personal reasons | | | | | | | | |
| 7. | Did you get contacted or assisted by medical team members: - Yes  No  IF yes, Medium of contact: -Telephone/WhatsApp/Email/video consult/others:…………………… | | | | | | | | |

| Medical Complications | | | | | | | | | | | | | | | | |
| --- | --- | --- | --- | --- | --- | --- | --- | --- | --- | --- | --- | --- | --- | --- | --- | --- |
| 1. | Symptoms of scleroderma worsened due to pandemic (can tick multiple) | | | | | Raynaud’s: | | | | | | Yes | | | No | |
|  |  |  |  |  |  | Skin tightening | | | | | | Yes | | | No | |
|  |  |  |  |  |  | joint pains | | | | | | Yes | | | No | |
|  |  |  |  |  |  | Any another:…………………………. | | | | | | | | | | |
| 2. | Hospital visit due to elevated disease | | | | | | | | | | | Yes | | | No | |
| 3. | Hospitalized during pandemic | | | | | | | | | | | Yes | | | No | |
| COVID related symptoms and other details | | | | | | | | | | | | | | | | |
| 1 | Developed any symptoms suggestive of COVID | | | | | Fever | | | | | | Yes | | | No | |
|  |  |  |  |  |  | Breathlessness | | | | | | Yes | | | No | |
|  |  |  |  |  |  | Anosmia | | | | | | Yes | | | No | |
|  |  |  |  |  |  | Diarrhea | | | | | | Yes | | | No | |
|  |  |  |  |  |  | Cough | | | | | | Yes | | | No | |
| 2 | Tested for novel Corona virus | | Yes   \| RT-PCR \| Antigen \| Result: ____ \| \| --- \| --- \| --- \| | | | | | | | | | | | | No | |
| 3. | Mingled with COVID positive patient | | | | | | | | | | | | Yes | | No | |
| 4. | Family member got COVID positive?  Yes  No  If Yes, Relationship with the COVID patient:……………………………………………….. | | | | | | | | | | | | | | | |
| 5. | Family member got exposed with COVID patient | | | | | | | | | | | | Yes | | No | |
| 6. | Mingled with people with flu | | | | | | | | | | | | Yes | | No | |
| Preventive Measures taken against COVID infection | | | | | | | | | | | | | | | | |
| 1. | Precaution by social distancing | | | | | | | | | | | | Yes | | No | |
| 2. | Handwash | | | | | | | | | | | | Yes | | No | |
| 3. | Isolating yourself | | | | | | | | | | | | Yes | | No | |
| 4. | Wearing masks | | | | | | | | | | | | Yes | | No | |
| 5. | Avoid going out to shops/or any other needs | | | | | | | | | | | | Yes | | No | |
| 6. | Taking bath immediately aftercoming back from outside | | | | | | | | | | | | Yes | | No | |
| 7. | Washing clothes separately of the person who goes out | | | | | | | | | | | | Yes | | No | |
| 8. | Taking homemade remedies like lemon juice, turmeric etc. | | | | | | | | | | | | Yes | | No | |
| 9. | Any COVID prophylaxis taken (Ayurveda/ allopathy/ homeopathy) | | | | | | | | | | | | Yes | | No | |
| 10. | Have you taken supplements | Zinc | | vitamin C | | | vitamin D | | | | Other:………………….. | | | | | |
| Psychosocial Issues | | | | | | | | | | | | | | | | |
| 1. | Loss of job due to pandemic | | | | | | | | | | | | | Yes | | No |
| 2. | Financial difficulty | | | | | | | | | | | | | Yes | | No |
| 3. | Money expended more than routine due to pandemic  Yes  No  If yes, approximate how much money expended more: - | | | | | | | | | | | | | | | |
| 4. | Finding difficulty to breath while wearing mask | | | | | | | | very much | | | | | mild | | No |
| 5. | Do you feel more vulnerable to COVID | | | | | | | | very much | | | | | mild | | No |
| 6. | Do you think your disease may flareup due to COVID | | | | | | | | very much | | | | | mild | | No |
| 7. | Do you think you’re more likely get infected with COVID | | | | | | | | very much | | | | | mild | | No |
| 8. | Why do you think that you may get infected | | | | Disease | | | Drug | | Others:……………………….. | | | | | | |

Patient Global assessment: On a scale of 0-10, how was your overall health in the last week?

0(good) 1 2 3 4 5 6 7 8 9 10(bad)

Hospital Anxiety and Depression Scale (HADS)

Instructions: Doctors are aware that emotions play an important part in most illnesses. If your doctor knows about these feelings, he or she will be able to help you more. This questionnaire is designed to help your doctor know how you feel. Read each item and circle the reply which comes closest to how you have been feeling in the past week. Don’t take too long over your replies: your immediate reaction to each item will probably be more accurate than a long thought out response.

Scleroderma Health Assessment Questionnaire

|  | Without Any difficulty (0) | With some difficulty (1) | With Much difficulty (2) | Cannot do at all  (3) |
| --- | --- | --- | --- | --- |
| Dressing & Grooming-are you able to   - Dressing yourself, including tying shoe lace and doing buttons? - Shampoo your hair? |  |  |  |  |
| Arising- are you able to   - Stand up from an armless straight chair? - Get in and out of bed? |  |  |  |  |
| Eating- are you able to   - Cut your meat? - Lift a full glass to your mouth? - Open a new milk carton |  |  |  |  |
| Walking -are you able to:   - Walk outdoors on flat ground - Climb up five stairs? |  |  |  |  |
| Hygiene-are you able to: -   - Wash outdoors on flat ground - Take a tub bath - Get on and off the toilet |  |  |  |  |
| Reach -are you able to   - Reach and get down a 5-pound object (such as a bag of sugar) from just over your head? - Bend and pick up clothing off the floor? |  |  |  |  |
| Grip -Are you able to:   - Open car doors? - Open jars that have been previously opened/ - Turn faucets on and off? |  |  |  |  |
| Activities -Are you able to:   - Run errands and shop? - Get in and out of a care? - Do chores such as vacuuming or yardwork? |  |  |  |  |
| Please check any categories for which you usually need help from another person  Dressing and Grooming  Arising Eating  Walking  Hygiene  Reach  Errands and chores  Grip  None  Unknown | | | | |
| Please check any AIDS or DEVICES that you usually use for any of these activities  Dressing and grooming: button hook, long shoe horn etc.  Grip: Jar opener  Arising: Special or Built -up Chair  Other  Eating: Built -up or special utensils  None  Walking: Cane walker /Crutches/Wheelchair  Unknown  Raised toilet seat, Bathtub seat, Bathtub bar  Reach, Long -handled appliances  HAQ Score: …………………….. | | | | |
